# Effects of Forest Therapy on Health Promotion among Middle-Aged Women: Focusing on Physiological Indicators

**DOI:** 10.3390/ijerph17124348

**Published:** 2020-06-17

**Authors:** Bum-Jin Park, Chang-Seob Shin, Won-Sop Shin, Chung-Yeub Chung, Si-Hyung Lee, Dong-Jun Kim, Youn-Hee Kim, Chang-Eun Park

**Affiliations:** 1 Department of Environment and Forest Resources, Chungnam National University, Daejeon 34134, Korea; bjpark@cnu.ac.kr; 2 Department of Forest Science, Chungbuk National University, Cheongju, Chungbuk 28644, Korea; sinna@chungbuk.ac.kr (C.-S.S.); shinwon@chungbuk.ac.kr (W.-S.S.); 3 Institute of Mental Health, Seoul 03156, Korea; maumnews@gmail.com; 4 Healience Seonmaeul, Hongcheon, Gangwon 25104, Korea; happy@serotonin.or.kr; 5 Center for Contemplative Science, Korea Advanced Institute of Science and Technology, Daejeon 34141, Korea; 6 National Center for Forest Therapy, Gimcheon, Gyeongbuk 39695, Korea

**Keywords:** forest therapy, middle-aged women, health promotion, serotonin, vitamin D

## Abstract

Women experience more stress in middle age than in other life stages, and health in middle age is vital, because it influences the quality of life in old age. In this study, the effects of a forest therapy program on physiological changes in 53 middle-aged women (divided into two groups) who lived in the city were examined. One group participated in a three-day program in the forest, followed by three days in the city; the other group participated in a three-day program in the city, followed by three days in the forest. Forest experiments were conducted in a “healing forest,” and urban experiments were conducted near a university campus. Blood tests were performed to evaluate the physiological effects of forest therapy. Differences in serotonin levels and vitamin D levels were verified before and after the forest (experimental group) and urban (control group) programs through paired *t*-tests. Statistically significant increases in serotonin levels were noted for participants in the forest program; vitamin D levels also increased, but not by statistically significant values. The findings of this study verify that forest therapy programs promote health among middle-aged women, and may prevent disease and improve quality of life.

## 1. Introduction

People in modern society experience significant stress in their daily lives. Stress is triggered by certain subjects or situations we encounter [1]. Stress can also be beneficial to health if it lasts for a limited time and is at a manageable level. However, if stress exceeds the manageable level and persists to become chronic, it has adverse effects on health [2,3]. Lack of an effective coping strategy for stress leads to various diseases [4,5].

During middle age, physical aging progresses and diseases become chronic [6,7]. In particular, middle-aged women experience more stress than men due to menopausal symptoms [8]. Most middle-aged women experience chronic stress, leading to a decrease in immunity, which in turn adversely affects their health [9]. As health management in middle age determines the quality of the rest of life, effective health management in this period is strategically important [10].

Interest in the effects of forests in preventing diseases and promoting health is increasing [10]. Activities in forests are effective in reducing stress, and programs aimed at healing in forests are drawing attention in the field of preventive medicine [11,12,13,14,15,16,17,18,19,20,21,22,23,24,25,26,27,28].

Forest therapy refers to activities using various environmental factors of the forest to promote the health of the human body. Forests have many elements that comfort people, such as beautiful scenery, clean air, sunlight, sound, phytoncide, and anions. According to Kaplan’s attention restoration theory, the forest environment gives humans a comfortable sensation [13]. The Savannah Hypothesis by Orians and Heerwagen claims that humans feel comfortable and less stressed in the natural environment [12]. Ulrich’s stress reduction theory states that humans quickly recover from stress when in the natural environment [11]. Attention restoration theory, psycho-evolution theory, biophilia hypothesis, and topophilia hypothesis are the background theories of forest therapy. According to attention restoration theory, directed attention should be reduced to recover from mental fatigue. Nature can provide the recovery environment in which mental fatigue can be relieved as directed attention is naturally reduced. The conditions of such an environment are compatibility, a sense of being away, fascination, and extent, all of which characterize nature [13]. According to psycho-evolution theory, humans are nature-friendly and emotionally recover in nature, because humans evolved through their adaptation to the natural environment [11]. In addition, the biophilia hypothesis states that humans experience pleasant feelings in nature, because humans genetically have an attachment to nature and a homing instinct [29]. The topophilia hypothesis extends the biophilia hypothesis. Topophilia refers to attachment to a place formed by experience. That is, humans have an affiliation with nature acquired through learning. This hypothesis explains the interest and positive feelings of humans for not only living elements but also non-living components, such as water and stones [30].

In a forest therapy program, methodology that maximizes the healing effect of forests is employed [31]. Six methods are used in forest therapy programs: plant therapy, water therapy, diet, psychotherapy, climate therapy, and exercise therapy [32,33,34]. The psychotherapy method is organized around meditation. Forest therapy programs for relieving stress involve actively performing meditation while listening to the sound of wind, water, and birds. Some forest therapy programs consist only of meditation, including breathing meditation and walking meditation [35].

Many studies have been conducted on the health promotion effects of forest therapy. A comprehensive literature review was included the first report on forest therapy in 1901, which revealed that the treatment of mental and tuberculosis patients was better in the forest tent ward than in the general ward in New York’s Manhattan State Hospital [36]. The first study on forest therapy in South Korea investigated the effects of forest therapy programs on depression reduction [37]. It was followed by various studies regarding children [38,39], elementary school students [40], middle and high school students [41], college students [42,43], office workers [32], workers with emotional issues [44], adult males [45,46], single mothers [47], pregnant women [26], middle-aged women [48,49], menopausal women [50], alcoholics [51,52], and the elderly [53,54].

Forest therapy is effective in alleviating and preventing the symptoms of stress-related diseases [21]. Studies regarding forest therapy as a healthcare program have verified its effects based on physiological indicators. Walking in the forest decreases the heart rate and increases the HF (high frequency) component of HRV (heart rate variability) [46], and the pulse wave transmission rate decreases after a forest bath [55]. A forest therapy program lowers the heart rate [49], blood pressure, and pulse rate [56], as well as adrenaline and cortisol levels [57]. Moreover, after walking in the forest, the saliva cortisol level [58] and heart rate [59] decreases. After spending time in the forest, there is no difference in HRV, but blood pressure decreases [60], the white blood cell count increases, and blood cytokines decrease [61]. After 30 min of resting in the forest, blood pressure and pulse rate have decreased [62]. As shown in these studies, the heart rate, HRV, blood pressure, pulse rate, and cortisol level have been used as indicators to verify the physiological effects of forest therapy, although serotonin and vitamin D levels have been insufficient.

This study verifies the health promotion effects of forest therapy programs on middle-aged women aged 40 to 64 years. Thus, serotonin and vitamin D levels were measured with blood tests before and after participation in programs in the forest and city, and then compared.

## 2. Materials and Methods

### 2.1. Subjects

Middle age has been classified as 40–60 years by Levinson et al. [63] and 44–65 years by Bühler [64]. This study defined middle age as 40–64 years old, given the rate of decline in functions associated with the maximum biological reproductivity of women [65].

The subjects of this study were middle-aged women between 40 and 64 years who were living in cities. Hypertensive patients, pregnant women, patients who had been treated in hospitals in the last three months, smokers, and people who had suffered circulatory and allergic diseases were excluded. Fifty-three subjects were selected and divided into two groups. For the period October 10–15, 2017, one group participated in a three-day program in the forest, followed by a three-day program in the city, while the other group participated in a three-day program in the city followed by a three-day program in the forest. This type of design is widely applied in the forest therapy field [49,60,66,67,68].

To recruit subjects without bias, a nationwide advertisement using public media was used. In addition, ₩50,000 KRW and the yoga mat that the subject used during the program were offered to all participants who faithfully participated in and complied with all experiment schedules.

All subjects gave their written informed consent for inclusion before they participated in the study. The study was conducted in accordance with the Declaration of Helsinki, and the protocol was approved by the Institutional Review Board of Chungnam National University (201708-SB-020–01).

### 2.2. Study Sites

The study site in the forest was a private healing forest located in Hongcheon-gun, Gangwon-do, where the forest-to-land area ratio is the highest (82%) in South Korea. The study site in the city was W University in Yeongdeungpo-gu, the only district among the 25 districts in Seoul without a forest, except for street trees, within a radius of 1 km.

#### 2.2.1. Forest Site

**Figure 1:**
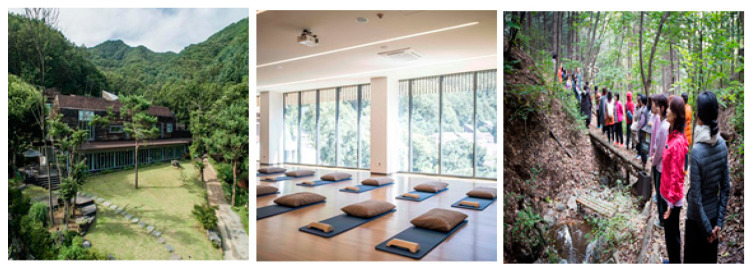
Forest site.

The forest site was located in Jungbangdae-ri, Seo-myeon, Hongcheon-gun, Gangwon-do, in the middle mountainous region, 250–580 m above sea level and covering an area of 747,800 m^2^. The site has many oak, pine, and nut pine trees, as well as abundant water. With respect to the distribution of species, Cork oak trees account for 53%; Mongolian oak, 15%; Japanese larch, 9%; nut pine, 8%; Cork oak-Mongolian oak, 7%; Cork oak-pine, 4%; Konara oak-pine, 2%; and Cork oak-pitch pine, 1%. There are 10 walking trails and a trekking course on a gentle slope. The degree of green naturality is grade 7 and above for approximately 80% of the area (Figure 1). The green naturality grade of 7 indicates an ecosystem in excellent status; the scenery is beautiful, with a distribution of major vegetation communities, and the natural environment of the site is of very high quality.

#### 2.2.2. Urban Site

**Figure 2:**
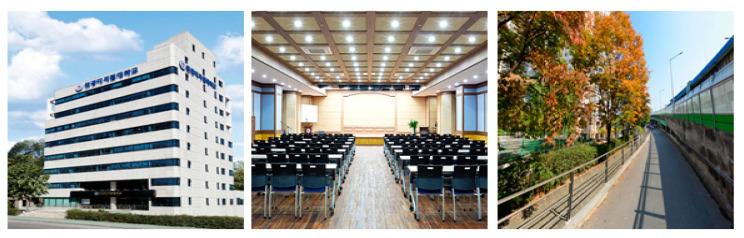
Urban site.

The urban site was the campus of W University in Daerim-dong, Yeongdeungpo-gu, Seoul. It has no green space within a radius of 1 km, and there is a sidewalk lined with trees along the roadway, which consists of ginkgo trees (Figure 2).

### 2.3. Measuring Tools

Serotonin and vitamin D levels were measured with a blood test.

Serotonin is a neurotransmitter that affects exercise, emotional regulation, and sleep [69]. Lack of serotonin increases depression, anxiety [70], and impulsivity [69], and sufficient levels of serotonin are conducive to comfortable, pleasant, and happy feelings. In this study, serotonin was selected as an indicator of mental health, in order to evaluate the differences in physiological changes with meditation and exercise in forest and urban environments.

Vitamin D is an essential nutrient that affects immune function. Vitamin D deficiencies have been reported to cause fractures and osteoporosis [71], metabolic syndrome [72], diabetes [73], cardiovascular disease [74], cancer [75], and depression [76,77,78]. In this study, vitamin D was also selected as an indicator of physical health to evaluate the differences in physiological changes with meditation and exercise in forest and urban environments.

Blood was collected before and after participation in the forest and urban programs by nurses managed by a medical specialist. The collected blood was put in a 10 mL anticoagulant tube and immediately delivered in an icebox to a specialized test laboratory.

To minimize the influence of external variables on the measurement results, alcohol intake was restricted for 12 h, and food intake and smoking were restricted for two hours before the blood test. Measurements were performed at the same time for each day of testing.

### 2.4. Program Setup and Progress

The program used in this study was developed by modifying the MBSR (mindfulness-based stress reduction) program of Dr. John Kabat-Zinn, which is a meditation program with verified effects. The main components of MBSR, which include body scan meditation, sitting meditation, mindful yoga practice, and walking meditation [79], were adopted to develop a program consisting of lying-down meditation, Seon yoga, serotonin walking, healing touch, stress relief meditation, and natural meditation (Table 1). The three-day program was guided by a professional instructor with more than three years of related experience.

The first day consisted of an orientation, agreement to the experiment, a preliminary survey, blood collection, lying-down meditation, and Seon yoga. Lying-down meditation is a relaxation technique: each subject lies down and relaxes the entire body. During Seon yoga, joint relaxation—which is easy, even for beginners—was performed while practicing yoga postures. The second day involved walking along a forest trail or a tree-lined street, experiencing a healing touch, lying down meditation, and stress relief meditation. Before walking on a forest trail or tree-lined street, the subjects relaxed their bodies by stretching, feeling their breath, and calming their minds. During healing touch, subjects formed pairs and massaged each other to relax tense muscles and accelerate blood circulation. Lying-down meditation was performed in the same way as on the first day, and stress relief meditation was done. Stress relief meditation involves practicing mindfulness to cope effectively with stress. The third day consisted of natural meditation to recover the five senses and rest by awakening one’s inner and outer bodies. After completing the schedule (Table 2 and Table 3), the subjects filled out a post-survey questionnaire and participated in blood collection.

### 2.5. Data Analysis

The data analysis was performed using the Statistical Package for the Social Sciences (SPSS) 21.0. A frequency analysis was performed to examine demographic characteristics of the subjects, and a paired *t*-test was performed to verify differences in physiological variables, such as the serotonin level and vitamin D level, before and after participating in the forest therapy program. The statistical significance level was set at *p* < 0.05.

## 3. Empirical Results

### 3.1. General Characteristics of Subjects

To examine the general characteristics of subjects (Table 4), a frequency analysis was conducted after classifying them by age, education level, residence area, household income, and occupation.

### 3.2. Physiological Effects of Forest Therapy

#### 3.2.1. Serotonin

**Figure 3:**
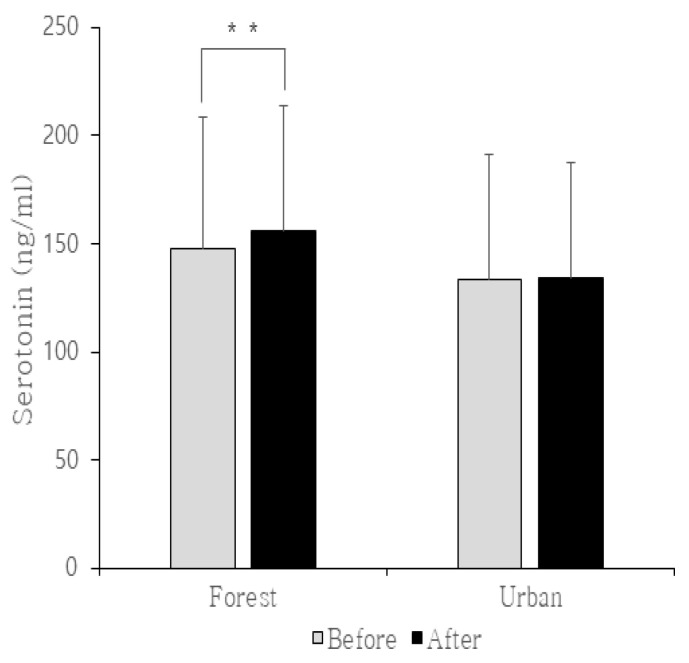
Changes in serotonin levels before and after forest therapy. ** indicates significant differences at *p* < 0.05.

The results of analyses using the paired *t*-test to verify changes in serotonin levels before and after forest therapy were as follows. The increase in the average serotonin level for the forest (experimental) group was statistically significant (from 147.62 ng/mL before therapy to 156.28 ng/mL after therapy (*p* < 0.05)). The average serotonin level for the urban (control) group increased from 133.52 ng/mL before therapy to 134.22 ng/mL after therapy, but the change was not statistically significant (*p* < 0.05) (Table 5; Figure 3).

#### 3.2.2. Vitamin D

**Figure 4:**
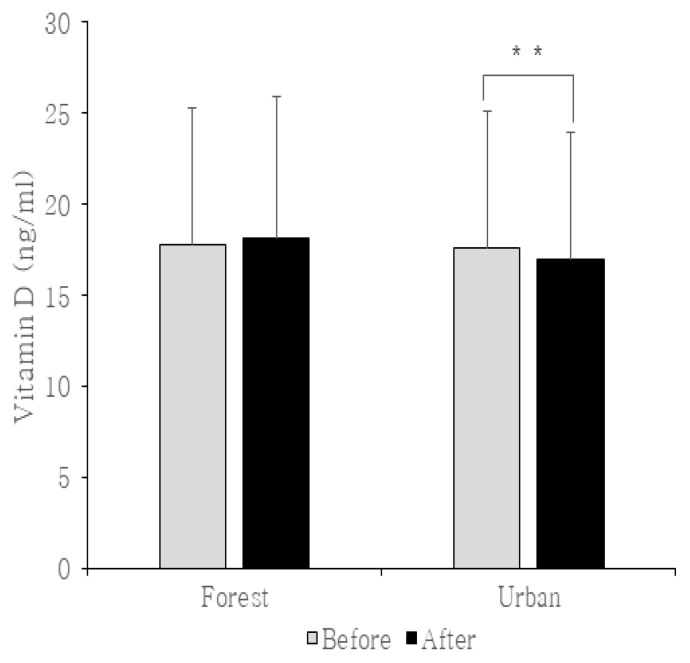
Changes in vitamin D levels before and after forest therapy. ** indicates significant differences at *p* < 0.05.

The results of analyses using a paired *t*-test to verify changes in vitamin D levels before and after forest therapy showed that on average, vitamin D levels increased from 17.81 ng/mL before therapy to 18.11 ng/mL after therapy in the forest (experimental) group; however, the change was not significant (*p* < 0.05). In the urban (control) group, the average vitamin D level significantly decreased from 17.62 ng/mL before therapy to 16.95 ng/mL after therapy (*p* < 0.05) (Table 6; Figure 4).

## 4. Discussion

This result was consistent with increased serotonin levels in older women who participated in a forest therapy program [54]. Serotonin is a neurotransmitter secreted from the hypothalamus of the brain. A low serotonin level can cause depression and anxiety and negatively affects sleep [69,70], and a high serotonin level can stimulate feelings of ease, comfort, and happiness [80].

Studies of the factors influencing the increase in serotonin levels found that the serotonin level increased after 15 weeks of Taekwondo training [81], short-term dance sports [82], track and field training [83], low-intensity swimming exercises [84], aerobic exercise on a treadmill for 90 min [85], and low-intensity exercise for 10 weeks [86]. Furthermore, a 60 min circulatory program with different types of exercise for stroke patients [87] and balance training for eight weeks [88] were found to be effective in increasing serotonin levels. Exercise increases the secretion of endorphins and serotonin [89]. Increased serotonin levels in the blood after exercise serves as an antidepressant [90]. However, another study found that high-intensity exercise reduced serotonin levels [91].

Findings from studies of factors besides exercise that contributed to increasing serotonin levels revealed that serotonin levels increased after a 12 week indoor yoga program for middle-aged women [92], a 20 week yoga program for adolescents with intellectual disabilities [93], and a 12 week yoga program for elderly patients with vascular dementia [94]. Serotonin increased after scent therapy was provided to older people [95]; sunlight also stimulated serotonin secretion [96].

Forest therapy is a complex therapy that uses the various environmental elements of forests. The forest therapy program developed in this study consisted of exercise, yoga, meditation, and touch, but mainly meditation.

Meditation calms and focuses the mind; the term was derived from the Latin word *meditatio*, meaning “deep thinking” [49]. Meditation activates the left frontal cortex. The activation of the left frontal cortex indicates absence of stress due to psychological stability. Meditation increases activities of the amygdala and hippocampus by stimulating the limbic system, a region of the brain that regulates emotion.

Meditation reduces stress hormones released due to stress. That is, the level of stress hormones, namely, cortisol, catecholamine [97], adrenocorticotropic hormone (ACTH) [98], and aldosterone [99], is reduced. Meanwhile, β-endorphin, which induces a positive state, and serotonin, which makes one feel happy, increases [100]. Meanwhile, NK (natural killer) cells have been more activated in a group that meditated in the forest compared with a group that had a walking exercise [101]. Furthermore, meditation also increases the release of serotonin by influencing neurotransmission to the cerebrum [97]. An increase in melatonin—a serotonin metabolite—in urine immediately after meditation was reported [101], and it has also been reported as increasing during meditation [102].

Meditation in the forest is effective because forest therapy factors assist with meditation [103]. Meditation is one of the psychotherapies used in forest therapy [34]. Meditation in the forest is comprised of feeling and understanding the vitality of nature through communion with nature. The effects of meditating in the forest are enhanced by forest therapy factors, such as the sound of water in a valley and the scent of pine trees [49].

In this study, the program administered to both the experimental and control groups was comprised of forms of meditation and exercise that have demonstrated an effect of increasing serotonin levels. Participation in the program by the control group (urban environment) did not affect their serotonin levels, while the experimental group (forest environment) showed increased serotonin levels. These results demonstrate that the meditation and exercise program was more effective in the forest environment than in the urban environment with respect to serotonin levels.

Since deficiencies of serotonin cause depression, anxiety, and other adverse conditions, a method using selective serotonin reuptake inhibitors (SSRIs) has been developed for therapeutic purposes; however, as the intake of SSRIs may have side effects, it is more effective to increase serotonin levels with a natural method, such as meditation, exercise, or sunlight exposure.

Vitamin D is an important factor for immunity, and its deficiency can cause metabolic syndrome [70], diabetes [73], cardiovascular disease [80], and cancer [75]. Furthermore, research shows that vitamin D deficiency can mentally increase the risk of depression [76,77,78,104].

In the case of the experimental group (forest environment), participation in the program did not affect the level of vitamin D in the body, but the vitamin D level decreased with program participation in the control group (urban environment). These results indicate that the meditation and exercise program in the forest environment inhibited a decrease in vitamin D levels compared to the meditation and exercise program in the urban environment.

## 5. Conclusions

The physiological effects of a forest therapy program for middle-aged women were measured using a blood test. The findings of this study revealed that forest therapy increased serotonin levels. It was verified that forest therapy was effective in promoting health by this action of serotonin. Increasing the serotonin level through the meditation-oriented forest therapy program developed in this study can contribute to illness prevention and improve quality of life.

The effects of the forest therapy program reported in this study can be considered as a combination of the effects of the forest environment and the program contents. An advantage of this study is that it provides the same program for the experimental group (forest environment) and the control group (urban environment), and compares the results before and after the program. This excludes the effect of the program, in order to objectively examine the effect of the forest environment on health. The results of this study strongly support the fact that modern people living in cities need not only to participate in programs involving meditation and exercise within their living environment, but also to make efforts to participate in programs offered in the natural environment.

The orders of the program processes differed for the experimental and control groups on the second day. This difference may cause bias, affecting the results. This point is a limitation of this study.

In the future, further research regarding people of different ages and with various diseases is needed. The results of this study can be used as evidential data for forest therapy.

## Figures and Tables

**Table 1:** List of items in the developed forest therapy program.

Program	Description of Content
Lying-down meditation	Meditation for fatigue relief and correct sleep habit building through relaxing meditation with deep rest, which relaxes the whole body while lying down
Seon yoga	Yoga consisting of actions to unwind joints that even beginners can easily follow
Serotonin walking	Walking that relaxes the body with stretching while appreciating the joy of walking and feeling one’s own breathing, leading to mental relaxation
Healing touch	Massage in pairs of participants that relieves muscle tension using props
Stress relief meditation	Meditation to relieve stress, understand the internal and external causes of stress, and practice mental habits for the effective prevention and relief of stress
Natural meditation	Meditation that facilitates the understanding of the inner and outer self through the five senses, leading to the restoration of the five senses and relaxation

**Table 2:** Forest therapy program schedule for the forest (experimental) group.

Time	Day 1	Day 2	Day 3
7:00–8:00 a.m.		Serotonin walking on a forest trail	
8:00–9:00 a.m.			
9:00–10:00 a.m.			9:30 Natural meditation
10:00–11:00 a.m.	Orientation		
11:00–12:00 a.m.	10:30 Questionnaire completion, blood collection		10:30 Questionnaire completion, blood collection
12:00–1:00 p.m.			
1:00–2:00 p.m.	1:30 p.m. Lying-down meditation	1:30 p.m. Lying-down meditation	
2:00–3:00 p.m.			
3:00–4:00 p.m.	Seon yoga	Healing touch	
4:00–5:00 p.m.			
5:00–6:00 p.m.			
6:00–7:00 p.m.			
7:00–8:00 p.m.			
8:00–9:00 p.m.		Stress relief meditation	

**Table 3:** Forest therapy program schedule for the urban (control) group.

Time	Day 1	Day 2	Day 3
7:00–8:00 a.m.			
8:00–9:00 a.m.			
9:00–10:00 a.m.			
10:00–11:00 a.m.	Orientation	Serotonin walking on a tree-lined street	10:30 Natural meditation
11:00–12:00 a.m.	10:30 Questionnaire completion, blood collection	Healing touch	11:30 Questionnaire completion, blood collection
12:00–1:00 p.m.			
1:00–2:00 p.m.	1:30 p.m. Lying-down meditation	1:30 p.m. Lying-down meditation	
2:00–3:00 p.m.	Seon yoga	Stress relief meditation	

**Table 4:** General characteristics of subjects.

Variables	Items	Number of Persons	%
Age	40–49	15	28
	50–59	27	51
	60–65	11	21
Education level	High-school graduate or lower	15	28
	College student	8	15
	College graduate	22	42
	Graduate student or higher	8	15
Household income (KRW)	Less than 2 million	6	11
	2 million–less than 3 million	7	13
	3 million–less than 4 million	10	19
	4 million–less than 5 million	9	17
	5 million–less than 6 million	5	9
	6 million or higher	16	30
Residence area	Seoul	25	47
	Capital area	23	43
	Provincial area	5	9
Occupation	Self-employed	6	11
	Office job	5	9
	Specialist	4	8
	Service industry job	3	6
	Full-time homemaker	28	53
	Other	7	13

**Table 5:** Changes in serotonin levels before and after forest therapy.

Variable	Before	After	*t*	*p*
	Mean (SD)	Mean (SD)		
Forest (*n* = 53)	147.62 (61.21)	156.28 (57.83)	−3.59 **	0.001
Urban (*n* = 53)	133.52 (57.72)	134.22 (53.40)	−0.44	0.67

** indicates significant differences at *p* < 0.05.

**Table 6:** Changes in vitamin D levels before and after forest therapy.

Variable	Before	After	*t*	*p*
	Mean (SD)	Mean (SD)		
Forest (n = 53)	17.81 (7.45)	18.11 (7.84)	−1.66	0.10
Urban (n = 53)	17.62 (7.45)	16.95 (7.02)	3.70 **	0.001

** indicates significant differences at *p* < 0.05.

## References

[1] Selye H. (1956). The Stress of Life.

[2] McEwen S. (2007). Physiology and neurobiology of stress and adaptation: Central role of the brain. Physiol. Rev..

[3] Hockey R., Hamilton V., Warburton A. (1979). Stress and the cognitive component of skilled performance. Human Stress and Cognition: An Information Processing Approach.

[4] Korte M., Koolhaas M., Wingfield C., McEwen S. (2005). The Darwinian concept of stress: Benefits of allostasis and costs of allostatic load and the trade-offs in health and disease. Neurosci. Biobehav. Rev..

[5] Bell M. (1977). Stressful life events and coping method in mental illness and illness behavior. Nurs. Res..

[6] Han K., Lee P., Lee Y. (2000). Influencing factors on symptoms of stress of middle aged women. J. Korean Acad. Nurs..

[7] Park J., Choi I. (2016). The Effect of K-MBSR program on stress, stress coping style, depression, anger and sleep of middle-aged women. J. Korean Acad. Nurs..

[8] Yeom J., Chun M. (2018). The effects of subjective health change on the marital satisfaction change of spouse in middle and old age: Focusing on gender difference. KJGSW.

[9] Trzesniewski K., Donnellan M., Robins R. (2003). Stability of self-esteem across the life span. J. Personal. Soc. Psychol..

[10] Park M., Kim K. (2014). Effects of yoga exercise program on response of stress, physical fitness and self-esteem in the middle-aged women. KJAN.

[11] Ulrich R. (1983). Aesthetic and affective response to natural environment. Behavior and the Natural Environment.

[12] Orians G., Penning-Rowsell E.C., Lowenthal D. (1986). An ecological and evolutionary approach to landscape aesthetics. Landscape Meanings and Values.

[13] Kaplan R., Kaplan S. (1989). The Experience of Nature: A Psychological Perspective.

[14] Baur J.W., Tynon J.F. (2010). Small-scale urban nature parks: Why should we care?. Leis. Sci..

[15] Heintzman P. (2009). Nature-based recreation and spirituality: A complex relationship. Leis. Sci..

[16] Miyazaki Y., Ikei H., Song C. (2014). Forest medicine research in Japan. Nihon Eiseigaku Zasshi.

[17] Li Q., Kobayashi M., Wakayama Y., Inagaki H., Katsumata M., Hirata Y., Hirata K., Shimizu T., Kawada T., Ohira T. (2009). Effect of phytoncide from trees on human natural killer function. Int. J. Immunopathol. Pharmacol..

[18] Li Q., Kawada T. (2011). Effect of forest therapy on the human psycho-neuro-endocrino-immune network. Nihon Eiseigaku Zasshi.

[19] Li Q., Kobayashi M., Inagaki H., Hirata Y., Li Y., Hirata K., Kawada T. (2010). A day trip to a forest park increases human natural killer activity and the expression of anti-cancer proteins in male subjects. J. Biol. Regul. Homeost. Agents.

[20] Ohira H., Takagi S., Masui K., Oishi M., Obata A. (1999). Effect of Shinrinyoku (forest-air bathing and walking): On mental and physical health (in Japanese). Bull. Tokai Women’s Coll..

[21] Park S., Woo J., Kim W., Lee Y. (2012). Sub-populations and disorders that can be applied to forest therapy. J. Korean Inst. For. Recreat..

[22] Yoo R., Jeong S. (2009). A Case study on application of the effect using forest on human health improvement and disease prevention. J. Korean Inst. For. Recreat..

[23] Park H., Shin C., Yeon P., Kim J. (2014). A comparative study on the stress recovery effect of forest therapy. J. Korean Inst. For. Recreat..

[24] Song J., Cha J., Lee C., Choi Y., Yeon P. (2014). Effects of forest healing program on stress response and spirituality in female nursing college students and their experience. J. Korean Inst. For. Recreat..

[25] Lee J., Yeon P., Park S., Kang J. (2018). Effects of forest therapy programs on the stress and emotional change of emotional labor workers. J. Korean Inst. For. Recreat..

[26] Park S., Yeon P., Hong C., Yeo E., Han S., Lee H., Kim Y. (2017). A Study on the effect of the forest healing programs on teachers’ stress and PANAS. Korean J. Environ. Ecol..

[27] Shin C., Yeon P., Kim Y., Um J., Im Y., Youn S., Lee S. (2015). The influence of a forest healing program on public servants in charge of social welfare and mental health care worker’s job stress and the profile of mood states (POMS). J. Korean For. Soc..

[28] Lee J. (2009). The Influence of forest scenes on psychophysiological responses. J. Korean For. Soc..

[29] Kellert R., Wilson O. (1993). The Biophilia Hypothesis.

[30] Beery T., Jönsson I., Elmberg J. (2015). From environmental connectedness to sustainable futures: Topophilia and human affiliation with nature. Sustainability.

[31] Kim Y., Kim D., Yeon P., Choi B. (2014). The Analysis of Interests and needs for the development of forest therapy program in adults. J. Korean Inst. For. Recreat..

[32] Park C., Kim D., Park K., Shin C., Kim Y. (2018). Effects of forest healing programs on resilience and happiness of employees. Korean J. Environ. Ecol..

[33] Chae Y., Kim J., Kang H. (2018). Literature review of forest healing therapy on Korean adults. J. Korean Biol. Nurs. Sci..

[34] Lee E., Park S., Yoo R., Hong S. (2011). Analysis on the activity contents of forest healing programs in Korea. J. Korean Inst. For. Recreat..

[35] Kim K. (2010). The effects of forest walking meditation using mindfulness paradigm on mental health in middle-age women. Korean J. Medit..

[36] Ahn H., Lee K. (2013). Towards a working model for an mbsr-informed forest healing program: Focusing on patients with hypertension. J. Korean Inst. For. Recreat..

[37] Shin W., Oh H. (1996). The influence of the forest program on depression level. J. Korean Inst. For. Recreat..

[38] Cho Y., Shin W., Yeon P. (2011). The influence of forest experience program length on sociality and psychology stability of children from low income families. J. Korean Inst. For. Recreat..

[39] Lee J., Hong J., Tae Y. (2017). Analysis of change of emotion and self-esteem of at-risk children through forest activities. J. Korean Inst. For. Recreat..

[40] Kim J., Shin C., Yeon P., Lee J., Kim M., Kim J., Yoo Y. (2013). Forest healing program impact on the mental health recovery of elementary school students. J. Korean Inst. For. Recreat..

[41] Oh K., Kim D., Kim J., Kim Y. (2016). The effects of forest-healing program on developing youth activity competence. Korean J. Youth Stud..

[42] Eom P., Whang M. (2015). Effects of Viewing Environments of Valley, forest road, and city on emotional state based on autonomic nervous system. J. Korean Inst. For. Recreat..

[43] Kim D., Lee S. (2014). Effects of forest therapy program in school forest on employment stress and anxiety of university students. PPE.

[44] Kim D., Kang H., Seo H. (2019). Qualitative analysis of emotional labor by forest healing. J. KOEN.

[45] Shin W., Yeon P., Lee J. (2007). The impact that a forest experience influences on a human mental state stability. J. Korean Inst. For. Recreat..

[46] Choi K., Shin W., Yeon P., Cho Y. (2011). The influence of forest walking exercise on human, stress and fatigue. J. Korean Inst. For. Recreat..

[47] Song J., Shin W., Yeon P., Choi M. (2009). The influence of forest therapeutic program on unmarried mothers’ depression and self-esteem. J. Korean For. Soc..

[48] Kim H., Lee Y., Koo C., Yeon P. (2016). The effect of emotional freedom technique (EFT) as forest therapy program on the menopause symptoms and the quality of life of the middle-aged women. J. Korean Inst. For. Recreat..

[49] Lee Y., Shin C. (2015). Effects of forest walking meditation on mood states and self-awareness in middle-aged women. J. Korean Inst. For. Recreat..

[50] Shin C., Yeon P., Cho M., Kim J. (2015). Effects of forest healing activity on women’s menopausal symptoms and mental health recovery. PPE.

[51] Yeon P. (2007). The relationships between forest experience and depression. J. Korean Inst. For. Recreat..

[52] Cha J., Kim S., Cheon D. (2016). Experiences of forest healing program among adult children of alcoholics. J. Korean Inst. For. Recreat..

[53] Choi J. (2011). Effects of forest exercise on the daily activity-related physical function and balance in the elderly. Korean J. Health Phys. Educ..

[54] Kim J., Shin C., Lee J. (2017). The Effects of forest healing program on mental health and melatonin of the elderly in the urban forest. PPE.

[55] Sin B., Lee K. (2018). The effects of forest bathing on social psychological and job stress. J. Naturop..

[56] Lee B., Lee H. (2013). Effects of occupational and social stresses after forest therapy. J. Naturop..

[57] Lee J., Park B., Tsunetsugu Y., Ohira T., Kagawa T., Miyazaki Y. (2011). Effect of forest bathing on physiological and psychological responses in young Japanese male subjects. Public Health.

[58] Olafsdottir G., Cloke P., Vögele C. (2017). Place, green exercise and stress: An exploration of lived experience and restorative effects. Health Place.

[59] Gidlow C., Jones M., Hurst G., Masterson D., Clark-Carter D., Tarvainen M., Nieuwenhuijsen M. (2016). Where to put your best foot forward: Psycho-physiological responses to walking in natural and urban environments. J. Environ. Psychol..

[60] Stigsdotter U., Corazon S., Sidenius U., Kristiansen J., Grahn P. (2017). It is not all bad for the grey city–A crossover study on physiological and psychological restoration in a forest and an urban environment. Health Place.

[61] Mao G., Lan X., Cao Y., Chen Z., He Z., Lv Y., Wang Y., Hu X., Wang G., Jing Y. (2012). Effects of short-term forest bathing on human health in a broad-leaved evergreen forest in Zhejiang Province, China. Biomed. Environ. Sci..

[62] Kjellgren A., Buhrkall H. (2010). A comparison of the restorative effect of a natural environment with that of a simulated natural environment. J. Environ. Psychol..

[63] Levinson D. (1986). A conception of adult development. Am. Psychol..

[64] Buhler C., Massarik F., Bugental J. (1968). The Course of Human Life.

[65] McLeod S.A., Erikson E. (2008). Psychosocial Stages Simply Psychology. http://www.simplypsychology.org/Erik-Erikson.html.

[66] Tsunetsugu Y., Park B.J., Ishii H., Hirano H., Kagawa T., Miyazaki Y. (2007). Physiological effects of Shinrin-yoku (taking in the atmosphere of the forest) in an old-growth broadleaf forest in Yamagata prefecture. Jpn. J. Physiol. Anthropol..

[67] Park B., Tsunetsugu Y., Kasetani T., Kagawa T., Miyazaki Y. (2010). The physiological effects of Shinrin-yoku (taking in the forest atmosphere or forest bathing): Evidence from field experiments in 24 forests across. Jpn. Environ. Health Prev. Med..

[68] Song C., Joung D., Ikei H., Igarashi M., Aga M., Park B., Miyazaki Y. (2013). Physiological and psychological effects of walking on young males in urban parks in winter. J. Physiol. Anthropol..

[69] Zhou F., Sari Y., Zhang J., Goodlett C., Li T. (2001). Prenatal alcohol exposure retards the migration and development of serotonin neurons in fetal C57BL mice. Dev. Brain Res..

[70] Svenningsson P., Chergui K., Rachleff I., Flajolet M., Zhang X., El Yacoubi M., Vaugeois J., Nomikos G., Greengard P. (2006). Alterations in 5-HT1B receptor function by p11 in depression-like states. Science.

[71] Holick M. (2006). Resurrection of vitamin D deficiency and rickets. J. Clin. Investig..

[72] Ford E., Ajani U., McGuire L., Liu S. (2005). Concentrations of serum vitamin D and the metabolic syndrome among U.S. adults. Diabetes Care.

[73] Mitri J., Muraru M., Pittas A. (2011). Vitamin D and type 2 diabetes: A systematic review. Eur. J. Clin. Nutr..

[74] Sun Q., Shi L., Rimm E., Giovannucci E., Hu F., Manson J., Rexrode K. (2011). Vitamin D intake and risk of cardiovascular disease in US men and women. Am. J. Clin. Nutr..

[75] Giovannucci E. (2019). Vitamin D and cancer incidence in the Harvard cohorts. Ann. Epidemiol..

[76] Ganji V., Milone C., Cody M., McCarty F., Wang Y. (2010). Serum vitamin D concentrations are related to depression in young adult US population: The Third National Health and Nutrition Examination Survey. Int. Arch. Med..

[77] Milaneschi Y., Shardell M., Corsi A., Vazzana R., Bandinelli S., Guralnik J., Ferrucci L. (2010). Serum 25-hydroxyvitamin D and depressive symptoms in older women and men. J. Clin. Endocrinol. Metab..

[78] Stewart R., Hirani V. (2010). Relationship between vitamin D levels and depressive symptoms in older residents from a national survey population. Psychosom. Med..

[79] Kabat-Zinn J. (2012). Mindfulness for Beginners: Reclaiming the Present Moment and Your Life.

[80] Bondy B., Erfurth A., Jonge S., Kruger M., Meyer H. (2000). Possible association of the short allele of the serotonin transporter promoter gene polymorphism(5-HTTLPR) with violent suicided. Mol. Psychiatry.

[81] Nam S., Kim H., Park S. (2009). An influence of iong entraining of Taekwondo of middle-aged women on her dopamine, serotonin and stress hormone. Exerc. Sci..

[82] Cho E. (2011). The Effect of Dance Sport Exercise on Neurotransmitter and Concentration in Youth. Master’s Thesis.

[83] Soares J., Naffah-Mazzacoratti M., Canalheiro E. (1994). Increased serotonin levels in physically traind men. Braz. J. Med. Biol. Res..

[84] Barchas J.D., Freedman D. (1963). Brainamines. Biochem. Pharmacol..

[85] Romanowski W., Grabiec S. (1974). The role of serotonin in the mechanism of central fatigue. Acta Physiol. Pol..

[86] Hong S., Lee D., Lee G. (2018). The effects of low intensity muscle strengthening on norepinephrine and epinephrine serotonin level in stroke patients with depression or emotional incontinence. KSIM.

[87] Baek I., Kim B., Park K. (2012). The effect of circuit class training on the synthesis of central serotonin in people. J. Korean Soc. Phys. Med..

[88] Im D., Baek I., Yoon B., Park C. (2012). Effect of 8-weeks’ balance training on depression inventory and serotonin in individuals post-stroke. KSW.

[89] Yoon D., Park J., Cho S., Park M., Kim S., Choi J. (2005). Depressive symptomatology and metabolic syndrome in Korean women. J. Obes. Metab. Syndr..

[90] Wipfli B., Landers D., Nagoshi C., Ringenbach S. (2011). An examination of serotonin and psychological variables in the relationship between exercise and mental health. Scand. J. Med. Sci. Sports.

[91] Morgan W., Brown D., Raglin J. (1987). Psychological monitoring of overtraining and staleness. Br. J. Sports Med..

[92] Youn T., Kim H., Jung W., Lee M. (2017). Effects of 12 weeks of yoga training program on physical fitness and cardiorespiratory function in middle-aged women. Korean J. Health Phys. Educ..

[93] Kim H. (2007). Effects of 20 Weeks Yoga on Neurotransmitter in Educable Mentally Retarded Teenagers. Master’s Thesis.

[94] Yoo J. (2010). Effect of yoga exercise on blood pressure, physical fitness, and blood variables in elderly with vascular dementia. KJGD.

[95] An H., Kim I., Kim Y. (2014). Analysis of the effect on geriatric depression by aromatherapy. J. Soc. Occup. Ther. Aged Dement..

[96] Vyssoki B., Praschak-Rieder N., Sonneck G., Bluml V., Willeit M., Kasper S., Kapusta N. (2012). Effects of sunshine on suicide rates. Compr. Psychiatry.

[97] An J., Kim J. (2014). A brain-scientific interpretation of the mental and physical healing by the Buddhist meditation. Buddhism Res..

[98] Yang E., Seo S., Jeong K., Yoon H., Lee D., Hwang B. (2011). The effects of short-term meditation and walking exercise in a forest on blood pressure, heart rate, NK-cell and POMS. J. Kinesiol..

[99] Manocha R. (2000). Why meditation. Aust. Fam. Phys..

[100] Jones M. (2001). Changes in cytokine production in healthy subjects practicingGuolin Qigong: A pilot study. BMC Complement. Altern. Med..

[101] Walton G., Pugh D., Gelderloos P., Macrae P. (1995). Stress reduction and preventing hypertension. J. Altern. Complement. Med..

[102] Tooley G., Armstrong S., Norman T., Sali A. (2000). Acute increases in night-time plasma melatonin levels following a period of meditation. Biol. Psychol..

[103] Shin Y. (2012). Differences of Psychophysiological Effects between Meditative and Athletic Walking in a Forest and Gymnasium. Ph.D. Thesis.

[104] May H., Bair T., Lappe D., Anderson J., Horne B., Carlquist J., Muhlestein A. (2010). Association of vitamin D levels with incident depression among a general cardiovascular population. Am. Heart J..

